# Acid‐Responsive Inks via Shuttling in a Pseudorotaxane Complex

**DOI:** 10.1002/cplu.202500453

**Published:** 2025-08-20

**Authors:** Yihan Shi, Robert Plavan, Miguel A. Soto, Mark J. MacLachlan

**Affiliations:** ^1^ Department of Chemistry University of British Columbia 2036 Main Mall Vancouver BC V6T 1Z1 Canada; ^2^ Stewart Blusson Quantum Matter Institute University of British Columbia 2355 East Mall Vancouver BC V6T 1Z4 Canada; ^3^ WPI Nano Life Science Institute Kanazawa University Kanazawa 920–1192 Japan; ^4^ Bioproducts Institute University of British Columbia 2385 East Mall Vancouver BC V6T 1Z3 Canada

**Keywords:** cellulose nanocrystals, host‐guest systems, molecular recognition, supramolecular chemistry

## Abstract

Pseudorotaxanes are host‐guest complexes where a guest molecule threads through a ring‐shaped host via noncovalent interactions. If the guest has two recognition sites, the host can dynamically shuttle between them. These complexes enable stimulus‐responsive molecular shuttles, where a stimulus, such as pH change, can control the position of the host, affecting properties like color and solubility. In this study, a guest molecule (**Gc**) with two recognition sites—1,4‐dialkoxyphenylene and pH‐sensitive 1,5‐diaminonaphthalene—is synthesized. These sites interact strongly with cyclobis(paraquat‐*p*‐phenylene) (**CBPQT^4^^+^**), a cationic host. **CBPQT^4^^+^** binds to the neutral diaminonaphthalene group to produce a green solution. Upon protonation of the diaminonaphthalene group, the host shifts to the dialkoxyphenylene site, turning the solution light orange. This color‐switching behavior remains stable over multiple protonation‐deprotonation cycles. The pseudorotaxane complex can also be disassembled via slow solvent diffusion, allowing recovery of the **Gc** and **CBPQT^4^^+^** components. Additionally, cellulose nanocrystal films incorporating the **Gc**⊂**CBPQT^4^^+^** complex show similar green‐to‐orange color shifts, demonstrating their potential for information encryption applications.

## Graphical abstract

GA: An acid‐responsive pseudorotaxane enables the switching of host cyclobis(paraquat‐*p*‐phenylene) (**CBPQT^4^^+^**) between two different binding sites on the guest axle via π–π stacking and electrostatic repulsion to achieve reversible color changes. Embedding the system into cellulose nanocrystal matrices produces acid‐responsive films that have potential for pattern encryption. The host in this system can be recovered by slow solvent diffusion.

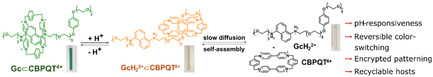

## 1 Introduction

Stimuli‐responsive materials have attracted much attention in recent years, particularly for their applications in fields such as drug delivery,^[^ ^1^, ^2^, ^3^, ^4^, ^5^ ^–^ ^6^ ^]^ sensing,^[^ ^2^ ^,^ ^7^, ^8^ ^–^ ^10^ ^]^ and encrypted messaging.^[^ ^9^ ^,^ ^11^, ^12^ ^–^ ^13^ ^]^ Supramolecular chemistry, involving the chemistry of weak intermolecular forces, is central to many of these materials. Guest binding events or stimulus‐induced structural changes have been harnessed as means to change the materials’ properties, such as color or fluorescence.^[^ ^14^ ^,^ ^15^ ^]^

Rotaxanes and pseudorotaxanes are fascinating molecular structures in the field of supramolecular chemistry. A rotaxane consists of a dumbbell‐shaped molecule, with two bulky groups at either end, and an encircling ring component that is mechanically trapped by the bulky groups. These bulky groups prevent the dissociation of the ring, and it has been demonstrated in only a few examples that the two components can be separated by, for example, the application of external force.^[^ ^16^ ^]^ In contrast, a pseudorotaxane has a similar structure but without the bulky groups, and thus the ring can dynamically associate/dissociate from the guest.^[^ ^17^ ^]^

Numerous examples of stimuli‐responsive rotaxanes have been reported. One of the most common stimuli used is pH because acid‐ and base‐sensitive groups can be easily incorporated into the structure of the many constituents of supramolecular assemblies.^[^ ^18^ ^]^ pH‐responsive rotaxanes incorporating pillar[n]arenes and crown ethers have been reported and these rotaxanes have shown potential as synthetic switches and shuttles.^[^ ^19^, ^20^ ^–^ ^21^ ^]^

**Figure 1:**
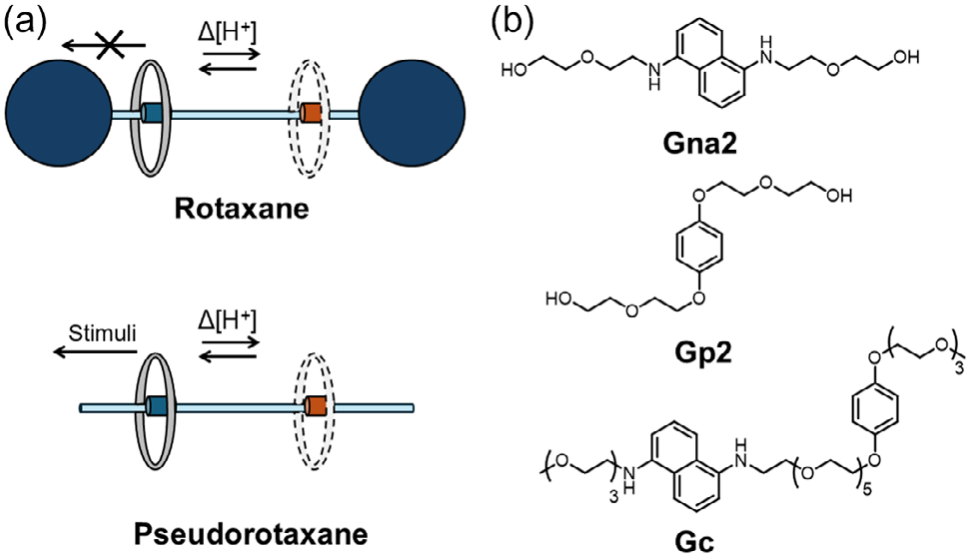
a) Illustration of host shuttling in a pH‐responsive rotaxane (top) and a pseudorotaxane (bottom). b) Structure of **Gc**, which incorporates the functional groups present in previously synthesized **Gna2** and **Gp2**.

Cyclobis(paraquat‐*p*‐phenylene) (**CBPQT** ^**4+**^) is a tetracationic, π‐electron deficient macrocycle with a cavity that can interact with small π‐electron rich moieties to form colorful rotaxanes, pseudorotaxanes and catenanes (mechanically‐linked rings).^[^ ^22^ ^]^ This host has also been used in the preparation of pH‐responsive structures that show relocation of the host on the guest's backbone after protonation/deprotonation cycles. For example, pH has been used to alter the electrostatic charge of guest molecules and thus tune their binding toward **CBPQT** ^**4+**^. This has been applied in the operation of switchable catenanes and other interlocked molecules.^[^ ^23^ ^]^ Similar examples have been reported in pseudorotaxane complexes, where a change in acid concentration causes threading and dethreading processes (**Figure** **1**a).^[^ ^24^, ^25^ ^–^ ^26^ ^]^

Cellulose nanocrystals (CNCs) are rod‐shaped nanoparticles obtained from the treatment of bulk cellulose with sulfuric acid.^[^ ^27^ ^]^ This process leaves sulfate half‐ester groups on the surface of the CNCs, imparting a negative charge that enables these nanoparticles to form a stable colloidal suspension in water due to the ion–ion repulsive interactions.^[^ ^28^ ^,^ ^29^ ^]^ When these interactions are minimized by adding salts (organic or inorganic), the suspension transitions into a gel. Our group has used this ion‐gelation approach to produce different CNC gels. We have demonstrated that host **CBPQT** ^**4+**^ gels CNCs to produce robust gels with active binding sites that can associate π‐electron‐rich molecules to form colorful host‐guest complexes.^[^ ^30^ ^]^ These complexes can disassociate to produce colorless gels under external stimuli. Drying these gels produced semitransparent films with **CBPQT** ^**4+**^ embedded; these materials show a comparable guest sorption performance to that observed in the parent gels.^[^ ^31^ ^]^ In our previous reports, we have explored various small aromatic guest molecules that allow for the tunability of host‐guest affinity and readable color in the material.^[^ ^30^ ^,^ ^31^ ^]^ Previous work proved that **CBPQT** ^**4+**^ could exchange between different small guest molecules to form acid/base‐switchable dynamic inks, but these inks could release guests into the surroundings during color‐switching, which might lead to unfavorable environmental pollution and irreversibility, thus limiting further applications.^[^ ^31^ ^]^

Here, we build upon our group's previous work by forming switchable colored pseudorotaxane complexes in a film embedded with **CBPQT** ^**4+**^. By design, the guest **Gc** can remain bound to the **CBPQT** ^**4+**^ both when it is protonated and deprotonated (Figure 1b).

## 2 Results and Discussion

### 2.1 Guest Synthesis

From our previous work, it was shown that 1,5‐diaminonaphthalene functionalized with oligoglycol chains (**Gna2**) can form pH‐responsive pseudorotaxanes with **CBPQT** ^**4+**^ while 1,4‐dihydroxybenzene functionalized with oligoglycol chains (**Gp2**) can form heat‐responsive pseudorotaxanes with **CBPQT** ^**4+**^. The association constants (*K*_a_) for **Gna2** and **Gp2** with **CBPQT** ^**4+**^ in solution are 42,600 and 1310 M^−1^, respectively (ethanol/water (v/v = 3:1), 25 °C).^[^ ^31^ ^]^ With this background knowledge in hand, we designed a new guest (**Gc**), shown in Figure 1b. This guest is expected to form a green pseudorotaxane with **CBPQT** ^**4+**^ sitting on the diaminonaphthalene (**DAN**) unit at neutral pH, considering the higher binding affinity of **DAN**. However, protonation of the system would cause the relocation of **CBPQT** ^**4+**^ from the dicationic form of **DAN** to the 1,4‐dialkoxybenzene (**DOB**) station, yielding a light orange complex.

**Scheme 1:**
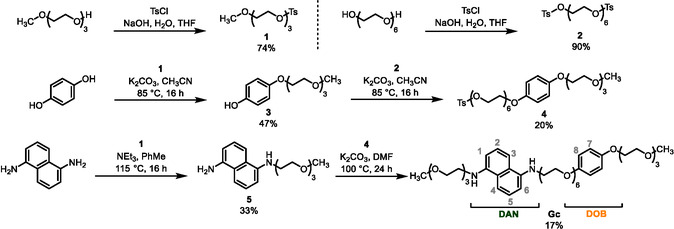
Synthesis of **Gc.**

**Gc** was synthesized following the steps shown in **Scheme** **1** (details in the Experimental Section). The structure of **Gc** was verified by ^1^H, ^13^C{^1^H} nuclear magnetic resonance (NMR) spectroscopy and mass spectrometry (see Supporting Information). For example, the ^1^H NMR spectrum of **Gc** recorded in CD_3_CN revealed the presence of the characteristic signals of protons belonging to the **DAN** and **DOB** components (δ_H2/5 _= 7.30 ppm, δ_H3/4 _= 7.24 ppm, δ_H1/6 _= 6.63 ppm and δ_H7/8 _= 6.85 ppm). Considering the guest solubility and CNC compatibility, we decided to use ethanol/water (v/v = 3:1) as the solvent in most cases. Once dissolved in ethanol/water (v/v = 3:1), solutions of both **Gc** and [**GcH**_**2**_]Cl_2_ (i.e. protonated **Gc**) appeared pale red.

### 2.2 UV–vis and NMR Characterization of Gc⊂CBPQT^4+^ and GcH_2_^2+^⊂CBPQT^4+^ Complex in Solution

**Figure 2:**
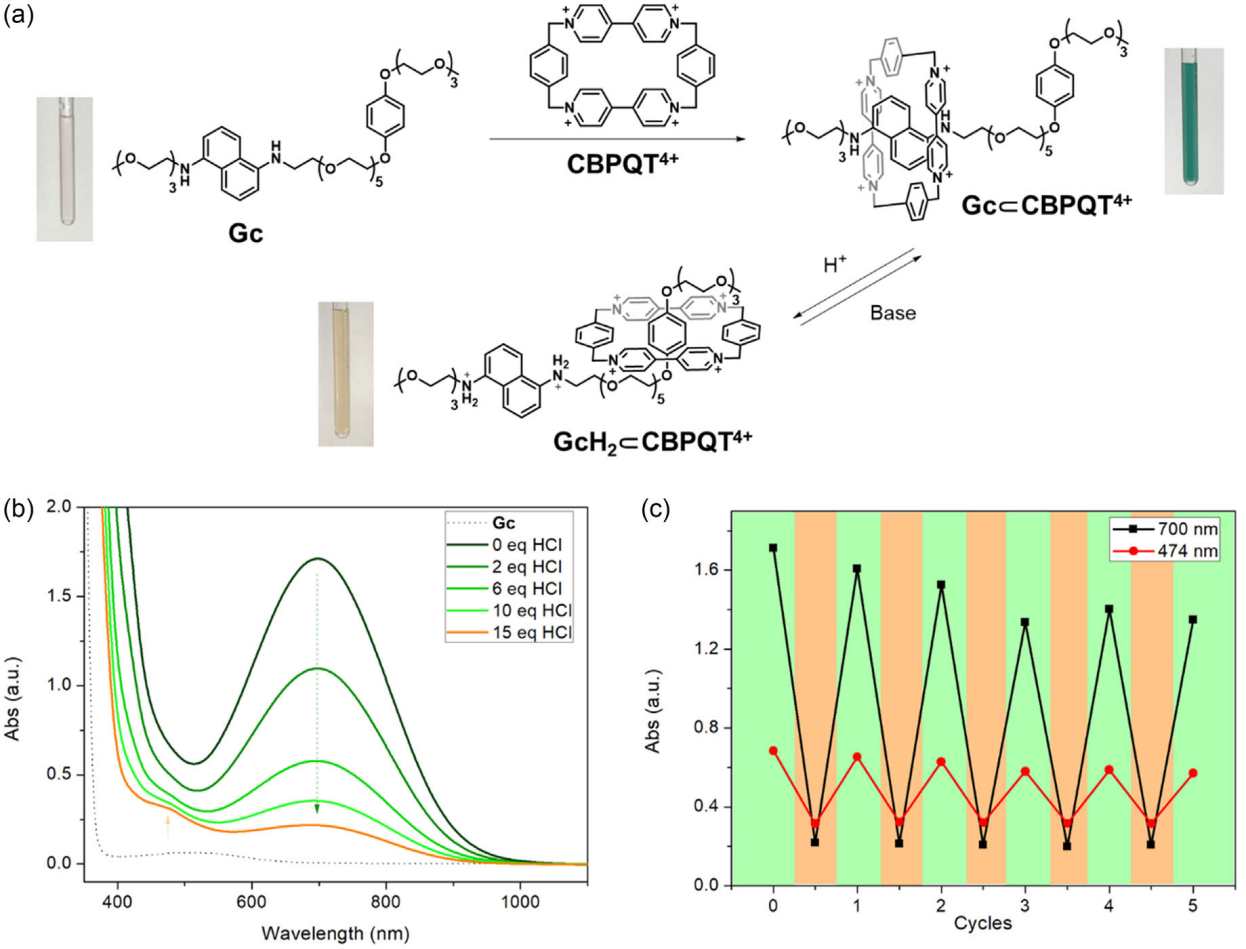
a) Illustration of the syntheses of **Gc⊂CBPQT** ^**4+**^ and **GcH**_**2**_^**2+**^ **⊂CBPQT** ^**4+**^ and photographs of their solutions. b) UV–vis titration of **Gc⊂CBPQT** ^**4+**^ in ethanol/water (v/v = 3:1) (3 mM). The addition of 2 M aqueous HCl leads to protonation of the **DAN** unit, resulting in a decrease in the CT band at 700 nm. c) Acid‐base switching between **Gc⊂CBPQT** ^**4+**^ (green) and **GcH**_**2**_^**2+**^ **⊂CBPQT** ^**4+**^ (orange) monitored by UV–vis spectroscopy for five cycles.

To study the properties of our host‐guest system, we first examined its visible color changes in solution. When 1 equiv. of **CBPQT**[Cl]_4_ was added to a solution of **Gc** (3 mM) in ethanol/water (v/v = 3:1), the solution immediately changed color from pale red to dark green (λ_max _= 700 nm) (**Figure** **2**a). This can be attributed to a **DAN⊂CBPQT** ^**4+**^ complex formed between **Gc** and **CBPQT** ^**4+**^ in the neutral solution.^[^ ^30^ ^]^ When 2 M HCl_(aq)_ was slowly added (up to 15 equiv. of H^+^), the band centered at 700 nm gradually decreased in intensity and a new band at 474 nm emerged (Figure 2b). The solution color changed from dark green to pale orange (Figure 2a); this change corresponds to 1) **DAN** protonation to disrupt **DAN⊂CBPQT** ^**4+**^ interactions and 2) formation of **DOB⊂CBPQT** ^**4+**^ interactions between **GcH**_**2**_^**2+**^ and **CBPQT** ^**4+**^ in acidic conditions (Figure 2a). The need for 15 equiv. of HCl for color conversion might be due to the low basicity of the aromatic amines in **DAN**. The stepwise addition of up to 15 equiv. of a NaOH solution (2 M) restored the solution to its original dark green color (Figure S14, Supporting Information), indicating the regeneration of **DAN⊂CBPQT** ^**4+**^ in neutral solution. While Figure 2a suggests that the ring slides along the guest axle, we cannot exclude the possibility that the **CBPQT** ^**4+**^ is fully dethreaded from the guest and recombines to form **DOB⊂CBPQT** ^**4+**^. The system is highly dynamic under the conditions of the experiment in solution.

The reproducibility of this acid‐base switch was tested for five cycles and tracked by the charge transfer (CT) bands (Figure 2c; details in Experimental Section). The **DAN⊂CBPQT** ^**4+**^ band at 700 nm decreased in the first two cycles and then reached a stable intensity while the **DOB⊂CBPQT** ^**4+**^ band intensity almost stayed the same. There are a few possible reasons for this decay. First, buildup of sodium chloride during the cycling will affect the ionic strength, and this may affect the photophysics. In particular, a buildup of Cl^−^ may affect the ion pairing of the **CBPQT** ^**4+**^. Second, decay might be due to some decomposition of one of the components. A third possible reason is that in the initial complex formation, **DAN** sites directly bind to free **CBPQT** ^**4+**^ cations. After protonation, most **CBPQT** ^**4+**^ associates with **DOB** sites, so during deprotonation, **DAN** sites compete dynamically with **DOB** sites to rebind the cavity of **CBPQT** ^**4+**^ cations, which seems to be less favorable than the first association. As a result, fewer **DOB** sites are bound by **CBPQT** ^**4+**^ in neutral solution after the first acid‐base cycle, leading to an absorbance decrease. After this cycle, the location of **CBPQT** ^**4+**^ on either **DAN** or **DOB** unit might reach an equilibrium so that the absorption intensity stabilized in the subsequent cycles.

The binding affinities of the **DAN** and **DOB** moieties were estimated in ethanol/water (v/v = 3:1) by titration of 0.3 mM **Gc** with 50 mM **CBPQT** ^**4+**^ solution and 3 mM **GcH**_**2**_^**2+**^ (**Gc** with 30 equiv. HCl) solution with 100 mM **CBPQT** ^**4+**^ solution, respectively; the titrations were monitored by UV–vis spectroscopy, and performed in triplicate (see details in Supporting Information).^[^ ^32^ ^]^ The association constant of **DAN** in **Gc** was obtained as 37,500 ± 7000 M^−1^, while the association constant of **DOB** in **GcH**_**2**_^**2+**^ was measured as 1103 ± 11 M^−1^. Compared with **Gna2**, **DAN** showed decreased binding affinity, which might be explained by the **DOB** unit competition during **Gc⊂CBPQT** ^**4+**^ complex formation. Similarly, the protonated **DAN** unit repulsion with **CBPQT** ^**4+**^ could also weaken the binding affinity of **DOB** in **GcH**_**2**_^**2+**^ compared to the association between **Gp2** and **CBPQT** ^**4+**^. However, the affinity difference is still large enough to achieve the proposed automatic host transfer from **DOB** to **DAN** in the **GcH**_**2**_^**2+**^ **⊂CBPQT** ^**4+**^ complex.

**Figure 3:**
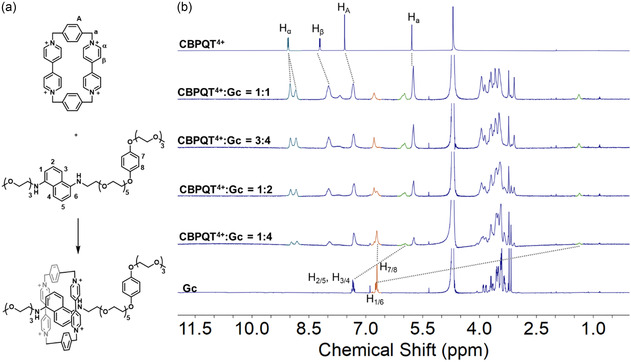
a) Illustration of the synthesis of **Gc⊂CBPQT** ^**4+**^. b) ^1^H NMR spectra (400 MHz, D_2_O, 25 °C) of **Gc**, **Gc⊂CBPQT** ^**4+**^ in different host‐guest ratios, and **CBPQT** ^4+^.

This pseudorotaxane formation process was also followed by NMR spectroscopic techniques. To further prove the formation of **DAN⊂CBPQT** ^**4+**^ and **DOB⊂CBPQT** ^**4+**^ during the acid‐base switching, a solution of **Gc** (3 mM in D_2_O) was titrated with **CBPQT** ^**4+**^ and analyzed by ^1^H NMR spectroscopy (**Figure** **3**a). The ^1^H NMR spectrum of a 1:1 mixture of **Gc** and **CBPQT** ^**4+**^ revealed significant shifts for the H_β_, and H_A_ protons of **CBPQT** ^**4+**^ and H_1/6_, H_2/5_, H_3/4_ protons (signals in green) of **DAN** on **Gc** (Figure 3b), along with signal splitting of H_α_ protons (resonances in blue) into two sets of resonances due to a slow motion of the naphthalene core within the host, which breaks the symmetry of **CBPQT** ^4+^.^[^ ^30^ ^]^ The observation of two sets of signals are the characteristics of **DAN⊂CBPQT** ^**4+**^ complexes, and all of these changes agree with reported results.^[^ ^31^ ^]^ Note that the solvent chosen for the NMR study (D_2_O) was different from the solvent used in the UV–vis titrations (ethanol/water, 3:1 v/v). This decision was made owing to the steep cost of deuterated ethanol and the expectation that the behavior would be sufficiently similar to allow the NMR data to qualitatively support the observations in the UV–vis experiments.

Furthermore, H_7/8_ protons (signals in orange) on the **DOB** unit showed no shifting but a decrease in intensity, meaning that some **DOB** units remained unassociated when the 1:1 **Gc⊂CBPQT** ^**4+**^ complex was formed. It is possible, however, that some **DOB** units interact with **CBPQT** ^**4+**^ because of the binding competition between **DAN** and **DOB**, so this could explain the subtle decrease in intensity for the corresponding resonances. The complex formed in a 1:1 host‐guest ratio was also analyzed by ^1^H‐^1^H nuclear Overhauser effect spectroscopy (NOESY) (Figure S16, Supporting Information). The correlations between split H_*α*_ protons proved the asymmetry and formation of **DAN⊂CBPQT** ^**4+**^.

For comparison, titration of **CBPQT** ^**4+**^ into a **Gc** solution (3 mM in D_2_O) with 30 equiv. of deuterium chloride (DCl) in D_2_O was performed and analyzed by ^1^H NMR spectroscopy (Figure S17, Supporting Information) to investigate the formation of the **GcD**_**2**_^**2+**^ **⊂CBPQT** ^**4+**^ complex. Resonance H_7/8_ (in orange) decreased in intensity immediately upon the addition of **CBPQT** ^**4+**^, while signals for H_1/6_, H_2/5_, and H_3/4_ remained unaltered. The resonances of **CBPQT** ^**4+**^ only shifted slightly during host addition and no signal splitting was observed when **GcD**_**2**_^**2+**^ **⊂CBPQT** ^**4+**^ complex was formed. All these changes in the NMR signals agree with the reported **DOB⊂CBPQT** ^**4+**^ complex and proved that no **DAN⊂CBPQT** ^**4+**^ interactions but **DOB⊂CBPQT** ^**4+**^ type interactions happened in the acidic environment.^[^ ^31^ ^]^

**Figure 4:**
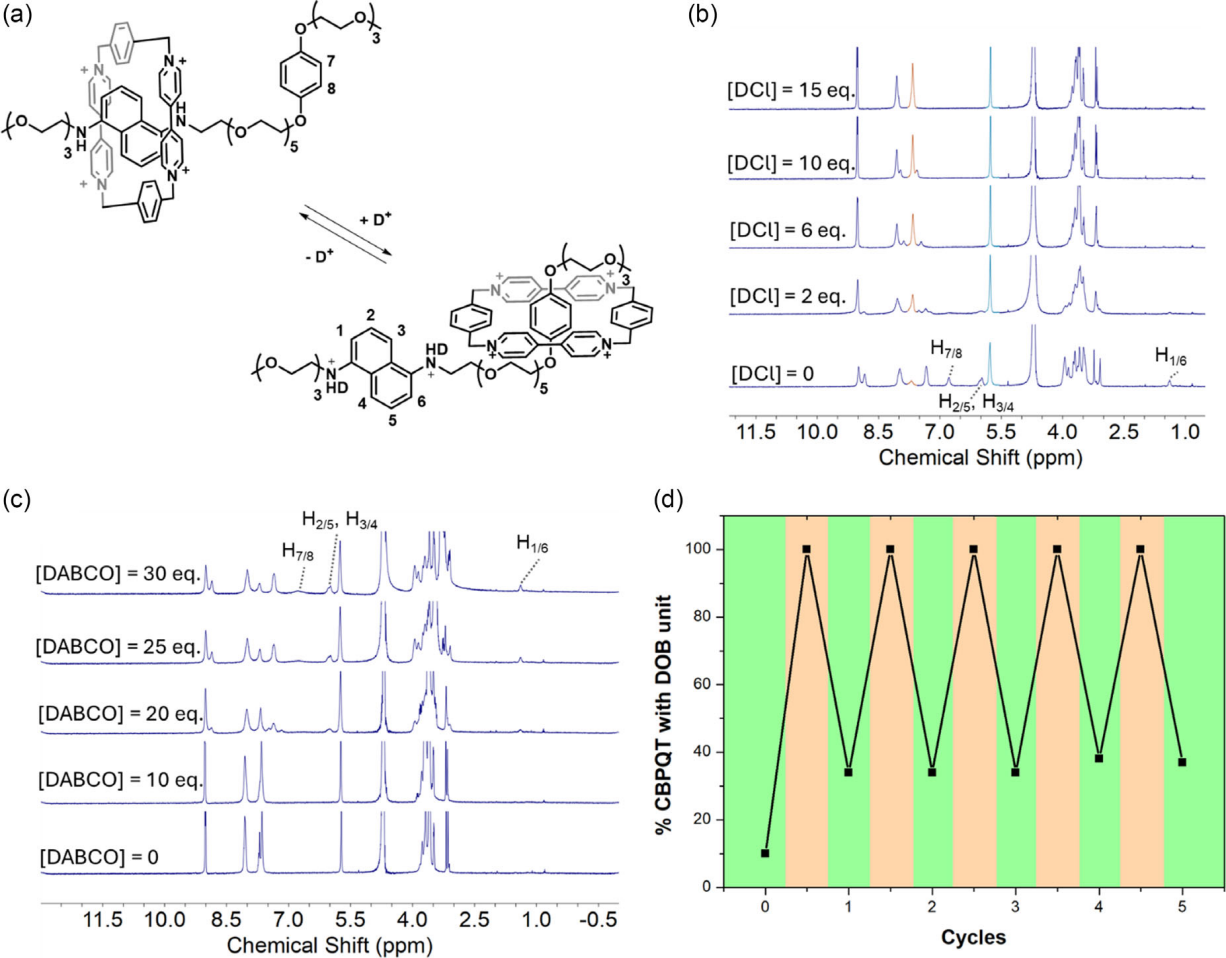
a) Illustration of the acid‐base switching between **Gc⊂CBPQT** ^**4+**^ and **GcD**_**2**_^**2+**^ **⊂CBPQT** ^**4+**^. b) ^1^H NMR titration (400 MHz, D_2_O, 25 °C) of **Gc⊂CBPQT** ^**4+**^ with DCl in D_2_O. c) ^1^H NMR titration (400 MHz, D_2_O, 25 °C) of **GcD**_**2**_^**2+**^ **⊂CBPQT** ^**4+**^ (with 30 equiv. DCl) with DABCO in D_2_O. d) Acid‐base switching between **Gc⊂CBPQT** ^**4+**^ (green) and **GcD**_**2**_^**2+**^ **⊂CBPQT** ^**4+**^ (orange) monitored by ^1^H NMR spectroscopy for five cycles.

Furthermore, the acid‐base titration was performed by adding DCl and 1,4‐diazabicyclo [2.2.2]octane (DABCO) as acid and base trigger, respectively, to better show the signal shifting during the protonation and deprotonation of **Gc⊂CBPQT** ^**4+**^ complex. The protonation process (**Figure** **4**a,b) upon adding DCl up to 15 equiv. showed: 1) the regeneration of unshifted H_1/6_, H_2/5_, H_3/4_ protons; and 2) the disappearance of H_7/8_ protons on the **DOB** unit along with the merging of a split H_*α*_ resonance. This relates well with the disassociation of **DAN⊂CBPQT** ^**4+**^ and association of the **DOB** unit with **CBPQT** ^**4+**^. The reverse process was observed upon deprotonation of **GcD**_**2**_^**2+**^ **⊂CBPQT** ^**4+**^ (Figure 4c). During the titration, the signal in orange shown between H_β_ and H_A_ at 7.68 ppm in neutral **Gc⊂CBPQT** ^**4+**^ was found to be a shifted H_A_ peak coming from **CBPQT** ^**4+**^ that associated with **DOB** sites in the neutral condition, indicating that not all **CBPQT** ^**4+**^ is interacting with **DAN** even in the first association step.

After the first acid‐base cycle, the integral of H_A_ (the signal at 7.68 ppm) increased because of the binding competition between **DAN** and **DOB** during the deprotonation process. Since proton H_a_ remains unchanged during the acid‐base cycle, calculating the integral ratio of H_A_ for **DOB⊂CBPQT** ^**4+**^ (shown in orange in Figure 4b) and H_a_ (shown in blue in Figure 4b) could allow one to estimate the percentage of occupied **DOB** in neutral and acidic solutions. In the beginning, only 10% of **CBPQT** ^**4+**^ reside on the **DOB** units. Upon protonation of the nitrogen atoms, 100% of **CBPQT** ^**4+**^ associated with **DOB** units. Titrating **GcD**_**2**_^**2+**^ **⊂CBPQT** ^**4+**^ with DABCO restored most of **DAN⊂CBPQT** ^**4+**^ interactions, but 34% of **CBPQT** ^**4+**^ remained associated with **DOB** units. By tracking the percentage of **DOB** occupied of **CBPQT** ^**4+**^, the reproducibility and stability of this acid‐base switch process was evaluated by performing five complete acid‐base cycles (Figure 4d and S18, Supporting Information; see details in experimental section). The percentage of **CBPQT** ^**4+**^ associated with **DOB** in **Gc⊂CBPQT** ^**4+**^ complex was found to increase from 10% to 34% after the first acid‐base cycle, but then remained relatively stable around 34% in the following four cycles, while the percentage of **CBPQT** ^**4+**^ associated with **DOB** in **GcD**_**2**_^**2+**^ **⊂CBPQT** ^**4+**^ complex was always 100%. These results match well with the UV–vis observations in Figure 2c, meaning that the binding mode of the **Gc⊂CBPQT** ^**4+**^ complex in both neutral and acidic states stabilized after the second acid‐base cycle.

As the binding affinity of **CBPQT** ^**4+**^ to **DAN** is higher than **DOB** (42,600 M^−1^ vs 1310M^−1^), the **CBPQT** ^**4+**^ molecule could be expected to transfer from **DOB** to **DAN** through the deprotonation of **GcH**_**2**_^**2+**^ **⊂CBPQT** ^**4+**^ complex (as long as there is no significant barrier to the translation process).^[^ ^31^ ^]^

### 2.3 Acid‐Base Cycling in CNC‐ CBPQT^4+^ Films

**Figure 5:**
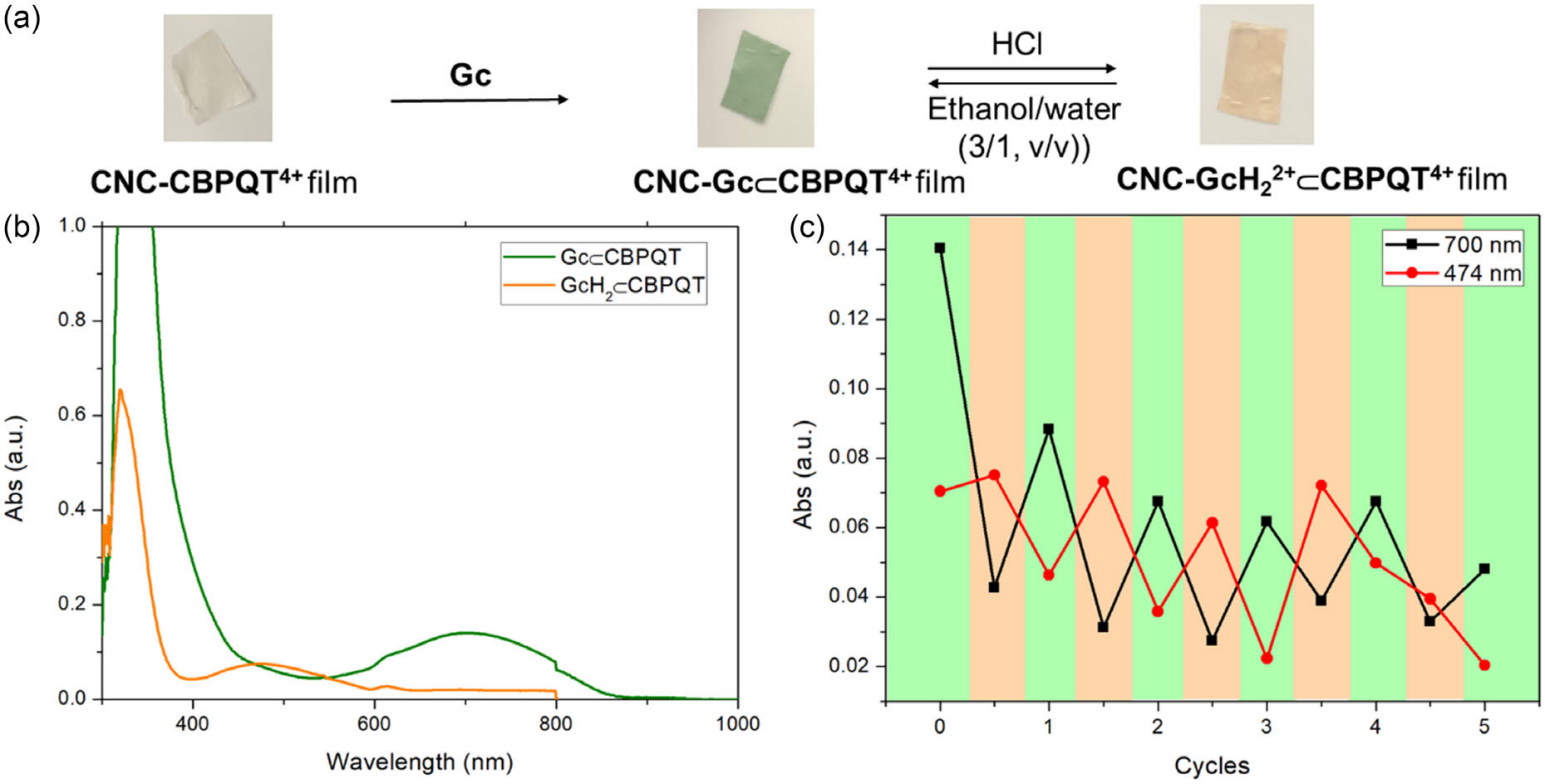
a) Illustration of the preparation of in‐film CNC‐**Gc⊂CBPQT** ^**4+**^, CNC‐**GcH**_**2**_^**2+**^ **⊂CBPQT** ^**4+**^ materials and their photographs. b) UV–vis spectra of CNC‐**Gc⊂CBPQT** ^**4+**^ film and CNC‐**GcH**_**2**_^**2+**^ **⊂CBPQT** ^**4+**^ film. c) Acid‐base switching between CNC‐**Gc⊂CBPQT** ^**4+**^ film (green) and CNC‐**GcH**_**2**_^**2+**^ **⊂CBPQT** ^**4+**^ film (orange) monitored by UV–vis spectroscopy for five cycles.

With solution‐based tests showing promising results, the next step was to investigate these properties in a CNC‐based material. We used a CNC‐**CBPQT** ^**4+**^ film, which has previously been demonstrated to successfully form switchable materials containing separated **DAN** and **DOB** molecules.^[^ ^30^ ^,^ ^31^ ^]^ Combining these moieties into a single molecule should produce responses like those observed in prior studies (**Figure** **5**a). When a CNC‐**CBPQT** ^**4+**^ film (0.5 cm × 1 cm) with 7 wt% **CBPQT** ^**4+**^ loading was immersed in a 20 mM **Gc** solution (ethanol/water (v/v = 3:1)) for 20 min, it gradually changed from colorless to green with a maximum absorbance at 700 nm (Figure 5b), corresponding to the formation of **Gc⊂CBPQT** ^**4+**^ complex, with the host residing on the **DAN** unit. Further immersion in a solution of HCl (ethanol/water (v/v = 4:1)), 400 mM) caused the film to transition to a pale orange color in about 2 min (using more diluted HCl (200 mM) extends this color‐switching process to 2 h). A CT band at 474 nm was detected in this film by UV–vis spectroscopy, which proved the generation of the **GcH**_**2**_^**2+**^ **⊂CBPQT** ^**4+**^ complex in the matrix (Figure 5b). The film was switched back to green by immersing it in the ethanol/water mixture (*v*/*v* = 3:1) for 15 min to let the film reach pH equilibrium with solvents. Base was avoided since the use of basic solution like Na_2_CO_3_ would give us a dark green film with irreversible color. The first deprotonation showed a much lower color intensity because simple dilution with ethanol and water would not cause full deprotonation (Figure 5c). However, when the protonation‐washing cycles were repeated, the absorbance corresponding to the in‐film complexes **Gc⊂CBPQT** ^**4+**^ and **GcH**_**2**_^**2+**^ **⊂CBPQT** ^**4+**^ stayed relatively stable after the second cycle. Starting from the fifth cycle, the absorbance of both films decreased, which might be explained by the instability of the film in the HCl solution; acidic conditions may cause hydrolysis and/or resuspension of the CNCs in the aqueous media. Using dilute HCl could possibly enhance the reproducibility of acid‐wash cycles.

As the objective of using a pseudorotaxane was to prevent guest release into the environment, the leakage of guests during each cycle was also investigated. After HCl and ethanol/water wash, the residual solutions were collected and combined. After air‐drying, the residue was redissolved in 500 μL D_2_O and analyzed by ^1^H NMR spectroscopy. For comparison, two additional CNC‐**CBPQT** ^**4+**^ films were immersed in **Gna2** and **Gp2** solutions, respectively, and underwent the same acid‐wash treatment as controls. The film with **Gna2** changed from green to colorless in the HCl solution and then stayed colorless in the ethanol/water mixture. The film with **Gp2** stayed orange during the acid‐wash cycle. Both collected washes were analyzed by ^1^H NMR spectroscopy (880 scans in 300 MHz). The spectra (Figure S19, Supporting Information) showed that the signals detected in the aromatic region (>6.0 ppm) from CNC‐**CBPQT** ^**4+**^ film with **Gc** are as small as the signals detected from the CNC‐**CBPQT** ^**4+**^ film with **Gp2**, which was expected to have almost no disassembly during the acid‐wash cycle. The film with **Gna2** released a higher proportion of the guest and host species compared to the other two tested films. In all three NMR spectra we observed signals in the 3.0–4.0 ppm region, which could correspond to acid‐hydrolyzed cellulose. All these results proved that combining **DOB** and **DAN** into a single guest molecule can improve the guest stability inside of pseudorotaxane complex during the pH switching process. Quantitative analysis of the guest leakage is still under investigation.

### 2.4 Applications

**Figure 6:**
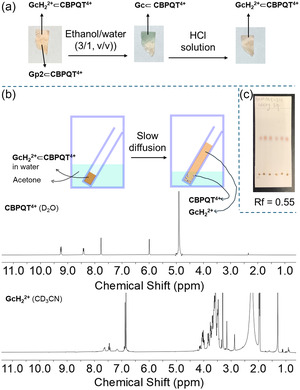
a) Photographs of a patterned CNC film, containing **Gc** as a dynamic ink. b) Illustration of host and guest separation by slow diffusion of acetone into **GcH**_**2**_^**2+**^ **⊂CBPQT** ^**4+**^ solution (solvent) with ^1^H NMR spectra (400 MHz, D_2_O/CD_3_CN, 25 °C) showing their composition. c) TLC plate showing a potential separation method to recover **Gc** (purple spot corresponding to **Gc** and the baseline spot corresponding to impurities).

Based on the pH‐responsiveness of the CNC‐**Gc⊂CBPQT** ^**4+**^ film, we designed a dynamic ink for pattern encryption and writing. Using 20 mM **Gc** solution (ethanol/water (v/v = 3:1)) as ink, we selectively colored an area on a CNC‐ **CBPQT** ^**4+**^ film. In a neutral pH, the area appeared green (CNC‐**Gc⊂CBPQT** ^**4+**^). Applying a 400 mM HCl solution to this area changed the pattern into orange (CNC‐**GcH**_**2**_^**2+**^ **⊂CBPQT** ^**4+**^). To encrypt this area/pattern/writing, a 20 mM **Gp2** solution (ethanol/water (v/v = 3:1)) was used to produce an orange background (CNC‐**Gp2⊂CBPQT** ^**4+**^) so that at the end, the entire film had an identical color with no visible pattern present (**Figure** **6**a). The UV–vis spectra showed that both regions share a similar CT band at 474 nm (Figure S20a, Supporting Information). Submerging the film into ethanol/water (v/v = 3:1) mixture revealed the green pattern with a CT band at 700 nm within 15 min (Figure S20b, Supporting Information). Using an HCl solution encrypted the pattern and reformed the all‐orange film. The use of the commercially available ethanol, water, and HCl solutions makes this encryption strategy applicable and end‐user accessible.

The **CBPQT** ^**4+**^ and **Gc** molecules could be separated and recycled from the host‐guest complexes. Slow diffusion of acetone into an aqueous solution of **GcH**_**2**_^**2+**^ **⊂CBPQT** ^**4+**^ produces the selective crystallization of **CBPQT** ^**4+**^ salt, leaving **Gc** in the aqueous solution (Figure 6b). ^1^H NMR spectroscopy of the recovered crystals in D_2_O showed no presence of **Gc** and only resonances of **CBPQT** ^**4+**^ were observed. On the other hand, the aqueous solution showed only **Gc** signals in the aromatic region. Thin‐layer chromatography (TLC, SiO_2_, dichloromethane/acetone (2:1)) was helpful to separate and recover **Gc** (Figure 6c). All of these proved that it is possible to recycle **CBPQT** ^**4+**^ and **Gc** molecules from **GcH**_**2**_^**2+**^ **⊂CBPQT** ^**4+**^ complexes. Ultra‐sonicating CNC matrices in solvents has the potential to disrupt the gel structure and dissolve the embedded **Gc⊂CBPQT** ^**4+**^ complexes into solution, giving a possible way to recycle the guest and host molecules from CNC‐based materials.^[^ ^33^ ^]^

## 3 Conclusion

We have synthesized a new π electron‐rich guest (**Gc**) bearing a pH‐sensitive 1,5‐diaminonaphthalene moiety and a 1,4‐dialkoxybenzene moiety. In solution, the guest **Gc** interacted with **CBPQT** ^**4+**^ to generate a green **Gc⊂CBPQT** ^**4+**^ pseudorotaxane, as confirmed by ^1^H NMR and UV–vis spectroscopic measurements. Interestingly, this complex can be protonated to give **GcH**_**2**_^**2+**^ **⊂CBPQT** ^**4+**^ complexes in acidic conditions, leading to a visual color change from green to orange with the guest remaining bound. Treating this with base regenerated **Gc⊂CBPQT** ^**4+**^ and the reversibility of the protonation‐deprotonation process was tested both in solution and inside cellulose nanocrystal films. Such pH‐reversible supramolecular assemblies can be used in a dynamic encryption strategy for patterns with almost no guest leakage and can be separated by slow diffusion to recycle host and guest molecules.

## 4 Experimental Section

4.1

4.1.1

##### Synthesis of Pseudorotaxane Guests

Guests **Gp2** (1,4‐bis[2‐(2‐hydroxyethoxy)ethoxy]benzene) and **Gna2** (1,5‐bis[2‐(2‐hydroxyethoxy)ethylamino]naphthalene), as well as compound **2** and the chloride salt of cyclobis(paraquat‐*p*‐phenylene) (**CBPQT** ^4+^), were synthesized following reported methodologies.^[^ ^34^ ^]^ In all cases, the spectroscopic characterization matched with the reported data. The syntheses of compounds **1**,^[^ ^35^ ^]^ **2**,^[^ ^36^ ^]^ **3**,^[^ ^37^ ^]^ **4**,^[^ ^37^ ^]^ **5**,^[^ ^38^ ^]^ and **Gc** ^[^ ^39^ ^]^ were adapted from literature procedures.

##### Synthesis of 1

Triethylene glycol monomethyl ether (22 mL, 139 mmol) dissolved in tetrahydrofuran (THF, 15 mL) was added dropwise to a solution of NaOH (8.1 g, 203 mmol) dissolved in THF (40 mL) and H_2_O (30 mL). The mixture was stirred at 0 °C for 1 h, followed by the dropwise addition (over 1 h) of a solution of *p*‐toluenesulfonyl chloride (24.9 g, 130 mmol) prepared in THF (50 mL). The reaction mixture was allowed to stir at room temperature for 3 h before quenching with H_2_O (20 mL) and extracting into DCM (150 mL). The combined organic layers were dried with MgSO_4_, filtered, and dried by rotary evaporation to yield a clear colorless oil (31 g, 188 mmol, 74%) identified as **1**. ^1^H NMR (300 MHz, CDCl_3_): δ = 2.44 (s, 3H), 3.37 (s, 3H), 3.51–3.74 (m, 10H), 4.16 (t, J = 5.0 Hz, 2H), 7.35 (d, J = 8.4 Hz, 2H), 7.81 (d, J = 8.4 Hz, 2H). ^13^C NMR (75 MHz, CDCl_3_): δ = 21.8, 59.2, 68.8, 69.4, 70.7, 70.9, 72.0, 128.1, 130.0, 133.3, 144.9 ppm. ESI (electrospray ionization)‐MS (mass spectrometry), *m/z*: found [**1** + Na]^+^ 341.0, calc. [**1** + Na]^+^ 341.4.

##### Synthesis of 2

Hexaethylene glycol (2.5 g, 8.9 mmol) was dissolved in 18 mL THF to prepare a clear solution. KOH (2.0 g, 35 mmol) was dissolved in 9 mL water and added into the THF solution. The solution was cooled to 0 °C with an ice bath. *p*‐Toluenesulfonyl chloride (4.1 g, 21 mmol) in THF (18 mL) was slowly added into the hexaethylene glycol/KOH solution in 2 h while keeping the temperature under 5 °C. After stirring for 5 h, the reaction was quenched with 18 mL of ice water. Ethyl acetate (3 × 20 mL) was used to extract the product from the solution. The organic layer was washed with 2 × 10 mL water, 2 × 10 mL brine solution and dried with MgSO_4_. Product **2** (4.5 g, 8.0 mmol, 90%) was isolated as a colorless oil by evaporating the solvent under vacuum. ^1^H NMR (CDCl_3_, 300 MHz, ppm): δ = 2.43 (s, 6H), 3.56–3.68 (m, 20H), 4.13 (m, 4H), 7.34 (d, J = 8.4 Hz, 4H), 7.79 (d, J = 8.4 Hz, 4H). ^13^C NMR (75 MHz, CDCl_3_): δ = 21.6, 68.7, 69.3, 70.5, 70.5, 70.6, 70.7, 128.0, 129.9, 133.0, 144.9 ppm. ESI‐MS, *m/z*: found [**2** + Na]^+^ 613.1, calc. [**2** + Na]^+^ 613.7.

##### Synthesis of 3

Compound **1** (14.42 g, 45.3 mmol) was added to a mixture of hydroquinone (10.0 g, 90.8 mmol) and K_2_CO_3_ (18.7 g, 135 mmol) in CH_3_CN (200 mL). The mixture was heated under reflux (90 °C) for 16 h before cooling it to room temperature. The mixture was filtered to remove residual K_2_CO_3_, and the solvent was removed by rotary evaporation. The crude product was purified by flash chromatography using an eluent gradient (SiO_2_, CH_3_Cl:MeOH = 100:1–40:1) to yield **3** as an orange oil (5.9 g, 23.0 mmol, 47%). ^1^H NMR (300 MHz, CDCl_3_): δ = 3.29 (s, 3H), 3.60 (m, 10H), 3.94 (t, 2H), 6.66 (s, 4H). ^13^C NMR (100 MHz, CD_3_Cl): δ = 59.1, 68.1, 70.0, 70.5, 70.7, 70.8, 71.9, 115.8, 116.1, 150.4, 152.5 ppm. ESI‐MS, *m/z*: found [**3** + H]^+^ 257.2, calc. [**3** + H]^+^ 257.3.

##### Synthesis of 4

Compound **2** (4.2 g, 7.1 mmol) in CH_3_CN (40 mL) was added to a mixture of compound **3** (0.92 g, 3.6 mmol) and K_2_CO_3_ (4.0 g, 29 mmol) in CH_3_CN (20 mL). The mixture was heated at reflux (85 °C) for 18 h and then cooled to room temperature. The mixture was filtered to remove residual K_2_CO_3_, and the solvent was removed by rotary evaporation. The crude product was purified by flash chromatography (R_f_ = 0.36) (SiO_2_, EtOAc:acetone = 7:1) yielding a yellow oil (472 mg, 0.70 mmol, 20%). ^1^H NMR (300 MHz, CDCl_3_): δ = 2.43 (s, 3H), 3.37 (s, 3H), 3.53–3.75 (m, 26H), 3.80–3.84 (t, J = 5.0 Hz, 4H), 4.05–4.16 (m, 6H), 6.82 (s, 4H), 7.34 (d, J = 8.1 Hz, 2H), 7.80 (d, J = 8.1 Hz, 2H). ^13^C NMR (100 MHz, CDCl_3_): δ = 21.7, 59.1, 68.1, 68.7, 69.3, 69.9, 70.6, 70.7, 70.8, 72.0, 115.6, 128.0, 129.9, 133.1, 144.9, 153.2 ppm. ESI‐MS, *m/z*: found [**4** + Na]^+^ 697.3, calc. [**4** + Na]^+^ 697.8.

##### Synthesis of 5

Compound **1** (8.3 g, 26.0 mmol) was added dropwise to a solution of 1,5‐diaminonaphthalene (3.4 g, 21.5  mmol) and triethylamine (10 mL) dissolved in toluene (100 mL). The mixture was heated at reflux (115 °C) for 16 h then was cooled to room temperature and dried by rotary evaporation. The crude product was dissolved in dichloromethane (DCM, 200 mL) and washed with aqueous saturated NaHCO_3_ (200 mL). The combined organic layers were dried with MgSO_4_, filtered, and dried by rotary evaporation. The crude product was further purified by flash chromatography using an eluent gradient (SiO_2_, DCM:acetone = 10:1 to 5:1) to yield **5** as a black oil. (2.2 g, 7.3 mmol, 33%). ^1^H NMR (400 MHz, CDCl_3_): δ = 3.37 (s, 3H), 3.44 (t, J = 5.2 Hz, 2H), 3.53–3.55 (m, 2H), 3.65–3.68 (m, 2H), 3.68–3.73 (m, 4H), 3.86 (t, J = 5.4 Hz, 2H), 6.62 (dd, J = 7.6 Hz, 0.8 Hz, 1H), 6.78 (dd, J = 7.2 Hz, 1.2 Hz, 1H), 7.18–7.24 (m, 2H), 7.30–7.35 (m, 2H). ^13^C NMR (100 MHz, CDCl_3_): δ = 44.1, 59.1, 69.4, 70.4, 70.6, 70.7, 72.0, 105.2, 110.4, 111.0, 112.0, 124.6, 125.1, 125.8, 143.8 ppm. ESI‐MS, *m/z*: found [**5** + H]^+^ 305.1, calc. [**5** + H]^+^ 305.4.

##### Synthesis of Gc

Compound **5** (146 mg, 0.5 mmol) in dimethylformamide (4 mL) was added to a mixture of compound **4** (472 mg, 0.7 mmol) and K_2_CO_3_ (109 mg, mmol). The mixture was heated at reflux (100 °C) for 23 h then cooled to room temperature. The reaction mixture was quenched with H_2_O (20 mL) and extracted into EtOAc (50 mL). The combined organic layers were dried with MgSO_4_, filtered, and dried by rotary evaporation. The crude product was further purified by flash chromatography using an eluent gradient (SiO_2_, DCM:acetone = 5:1 (R_f_ = 0.07) to 2:1 (R_f_ = 0.54)) to yield **Gc** as a red oil (66 mg, 0.08 mmol, 17%). ^1^H NMR (400 MHz, CD_3_CN): δ  = 3.29 (s, 3H), 3.31 (s, 3H), 3.41 (m, 4H), 3.48 (m, 4H), 3.50–3.77 (m, 32H), 3.80 (m, 4H), 4.03 (m, 4H), 6.63 (d, J = 7.2 Hz, 2H), 6.85 (s, 4H), 7.24 (d, J = 8.4 Hz, 2H), 7.30 (t, J = 7.8 Hz, 2H). ^13^C NMR (100 MHz, CD_3_CN): δ = 43.4, 57.9, 67.9, 68.9, 69.4, 70.0–70.3, 71.6, 104.1, 108.9, 115.4, 117.3, 124.0, 125.4, 144.2, 153.0 ppm. ESI‐HRMS (high‐resolution mass spectrometry), *m/z*: found [**Gc** + H]^+^ 807.4634, calc. [**Gc** + H]^+^ 807.4643.

## Supporting Information

Supporting information for this article is available on the WWW under https://doi.org/10.1002/cplu.202500453.

## Conflict of Interest

The authors declare no conflicts of interest.

## Supporting information

Supplementary Material

## Data Availability

The data that support the findings of this study are available in the supplementary material of this article.
