# Supplementary material for: Acid‐Responsive Inks via Shuttling in a Pseudorotaxane Complex
Source: Chempluschem. 2025 Aug 20;90(10):e202500453. doi: 10.1002/cplu.202500453 (PMC12509471; doi:10.1002/cplu.202500453)
Supplement: Supplementary file 1 — Supplementary Material [file CPLU-90-e202500453-s001.pdf]

Supplementary Information

Acid-Responsive Inks via Shuttling in a Pseudorotaxane Complex

Yihan Shi,[a] Robert Plavan,[a] Miguel A. Soto[a] and Mark J. MacLachlan*[abcd]

[a] *Department of Chemistry, University of British Columbia, 2036 Main Mall, Vancouver, British Columbia, V6T 1Z1 Canada*

[b] *Quantum Matter Institute, University of British Columbia, 2355 East Mall, Vancouver, British Columbia, V6T 1Z4 Canada*

[c] *WPI Nano Life Science Institute, Kanazawa University, Kanazawa, 920-1192 Japan*

[d] *Bioproducts Institute, University of British Columbia, 2385 East Mall, Vancouver, British Columbia, V6T 1Z3 Canada*

1. General

1,5-Diaminonaphthalene was purchased from TCI. Triethylamine, toluene, and acetone were purchased from Fisher. All other chemicals were purchased from Sigma Aldrich and were used without further purification unless otherwise mentioned. Flash chromatography columns were run with silica purchased from SiliCycle (230-400 mesh). Sodium-neutralized cellulose nanocrystals were purchased from CelluForce as an aqueous suspension (CNC-Na^+, 6.1 wt%, pH = 6.5). Deuterated solvents D_2O, CD_3CN and $CDCl_3$ were purchased from Cambridge Isotope Laboratories and Sigma-Aldrich. Deionized water was used throughout.

^1H, $^{13}C\{^1H\}$, COSY, and EXSY NMR spectroscopic data were obtained using either a Bruker Avance 300 MHz, a Bruker AV III HD 400 MHz, or a Bruker Avance 600 MHz NMR spectrometer. Chemical shifts are reported in parts per million (ppm) and referenced to residual protonated solvent. UV-vis spectra were measured using a Cary 5000 UV-vis-NIR spectrometer using 1.0 cm path-length quartz cuvettes. Mass spectrometry was performed using a Waters ZQ equipped with

ESCI ion source and an Agilent 6545 QTOF with ESI ion source. All photographs were taken with an iPhone XR.

2. Precursors and guest characterization

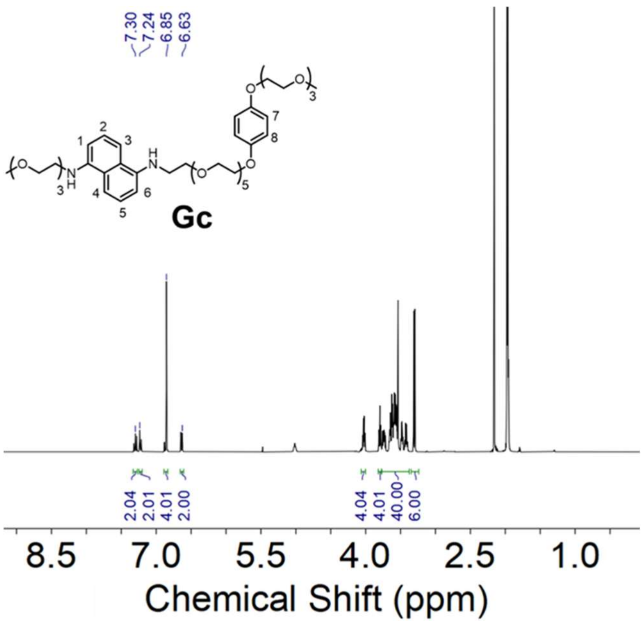

Figure S1. ^{1}H NMR spectrum (400 MHz, CD_3CN, 25 °C) of **Gc**.

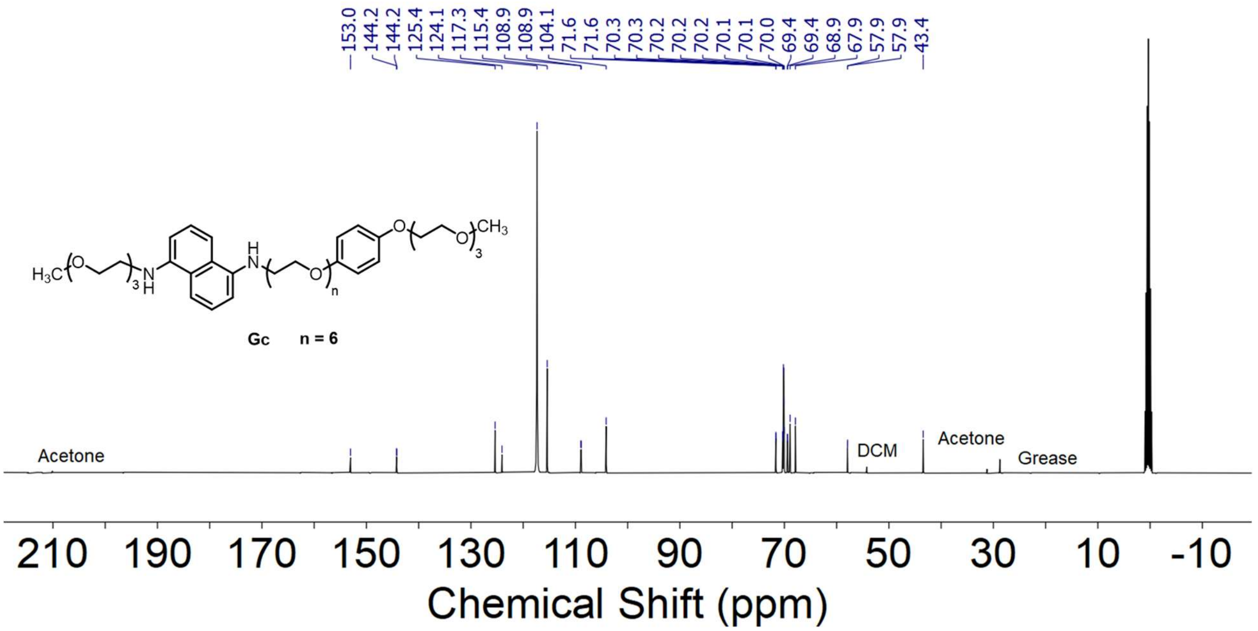

Figure S2. $^{13}C\{^{1}H\}$ NMR spectrum (100 MHz, CD_3CN, 25 °C) of **Gc**.

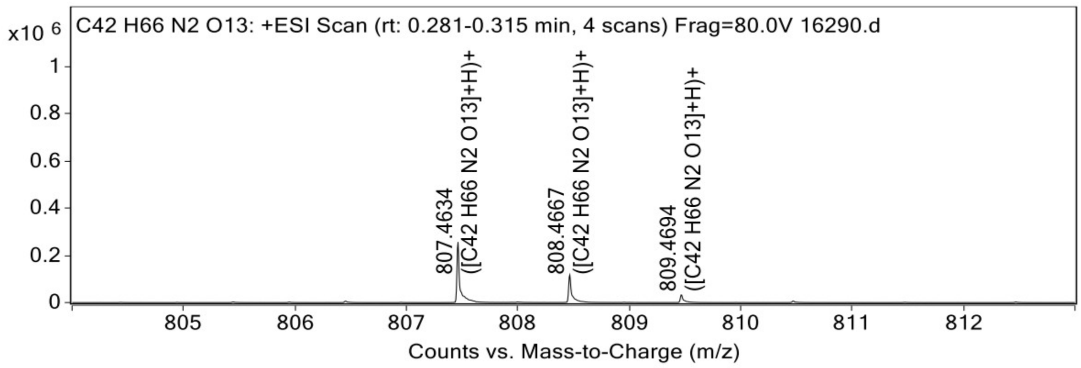

Figure S3. High-resolution mass spectrum (HR-MS) of **Gc**.

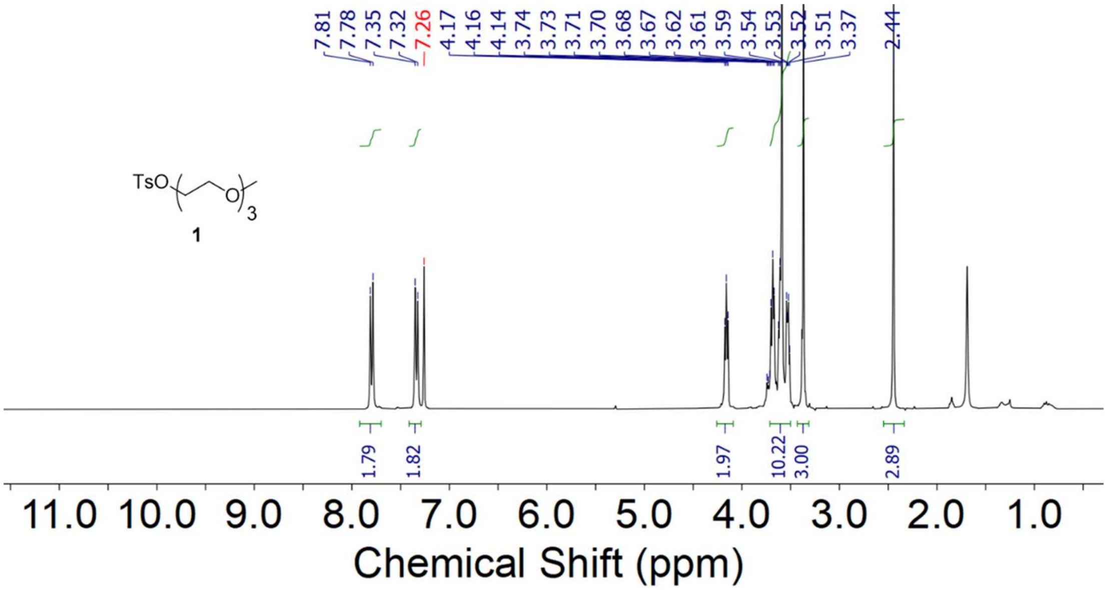

Figure S4. ^{1}H NMR spectrum (300 MHz, $CDCl_3$, 25 °C) of **1**.

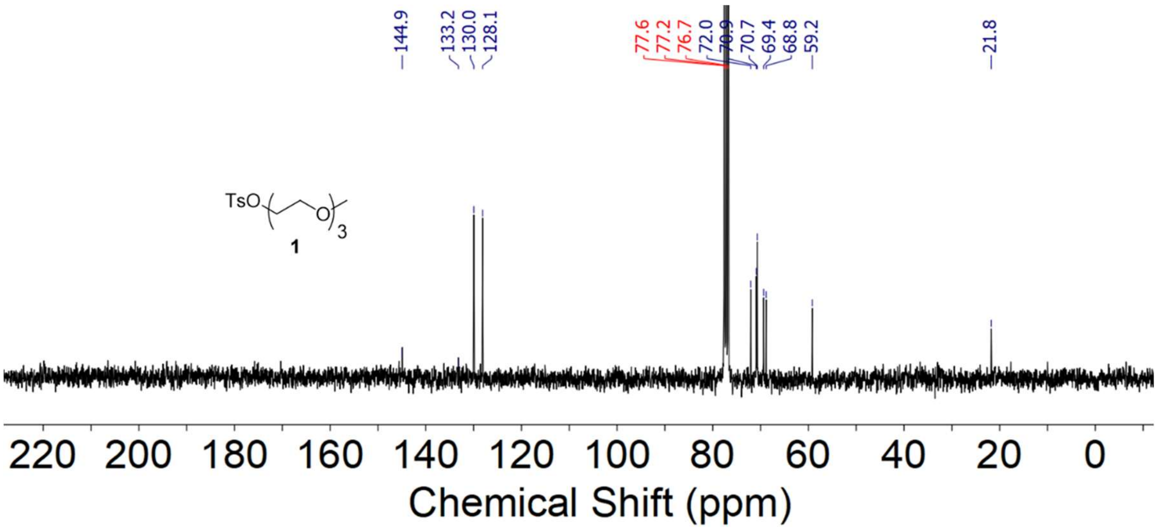

Figure S5. $^{13}C\{^{1}H\}$ NMR spectrum (75 MHz, $CDCl_3$, 25 °C) of **1**.

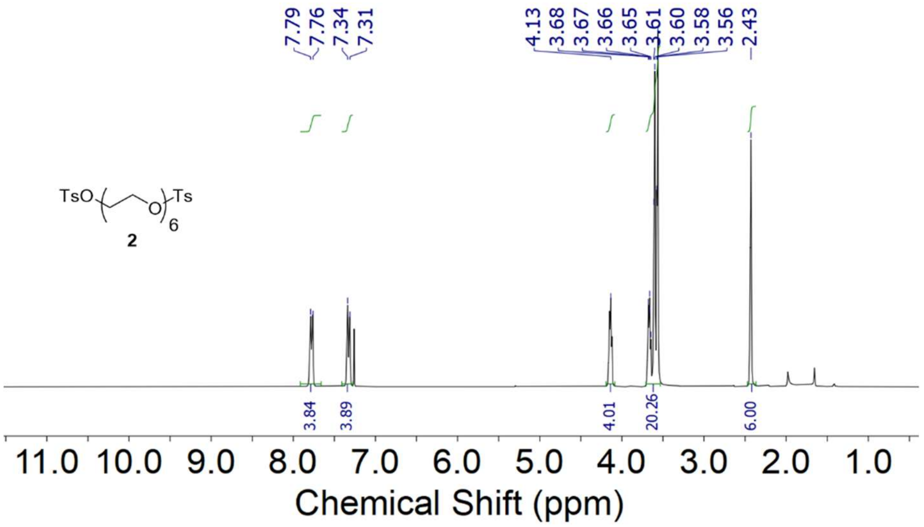

Figure S6. ^{1}H NMR spectrum (300 MHz, $CDCl_3$, 25 °C) of **2**.

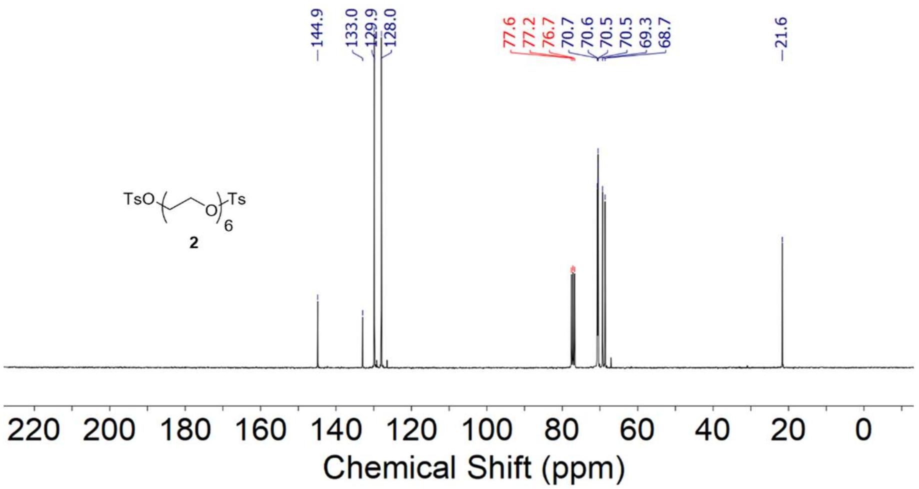

Figure S7. $^{13}C\{^{1}H\}$ NMR spectrum (75 MHz, $CDCl_3$, 25 °C) of **2**.

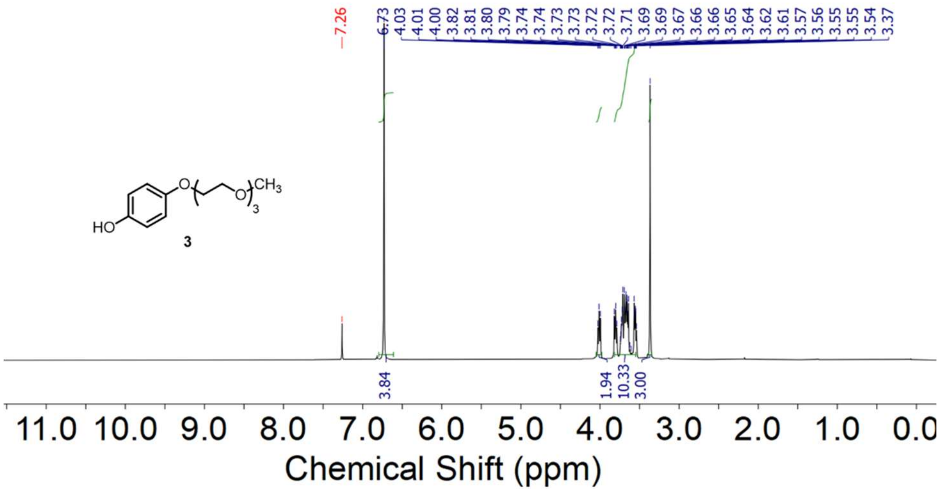

Figure S8. ^{1}H NMR spectrum (300 MHz, $CDCl_3$, 25 °C) of **3**.

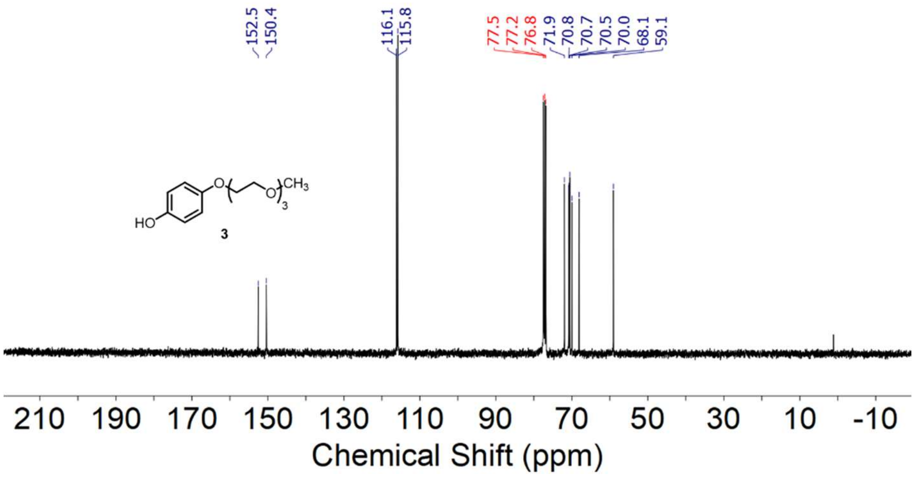

Figure S9. $^{13}C\{^1H\}$ NMR spectrum (100 MHz, $CDCl_3$, 25 °C) of **3**.

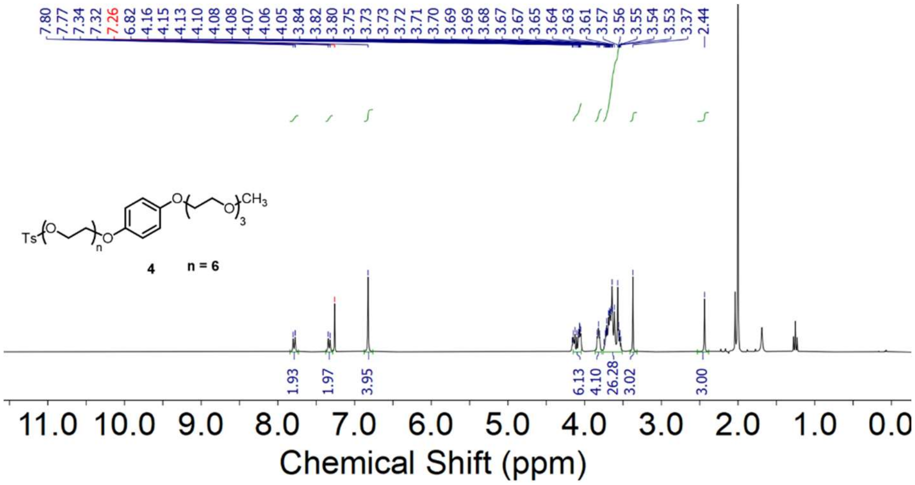

Figure S10. ^1H NMR spectrum (300 MHz, $CDCl_3$, 25 °C) of **4**.

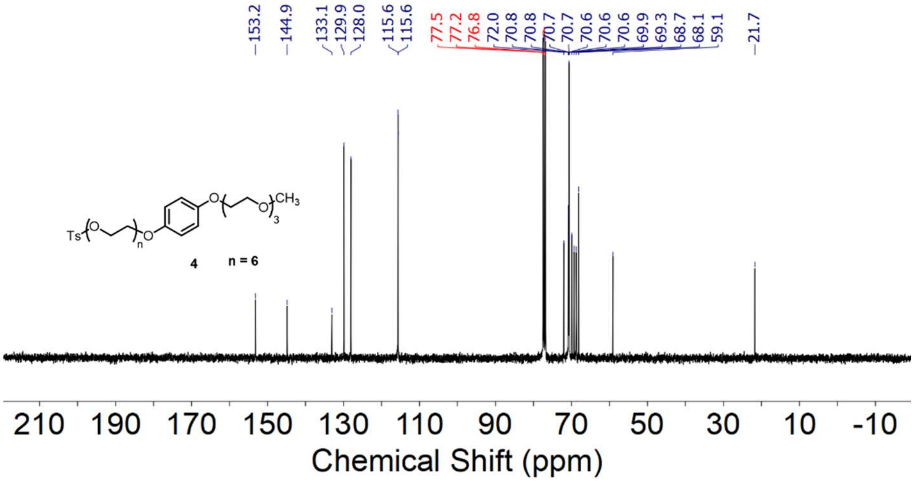

Figure S11. $^{13}C\{^1H\}$ NMR spectrum (100 MHz, $CDCl_3$, 25 °C) of **4**.

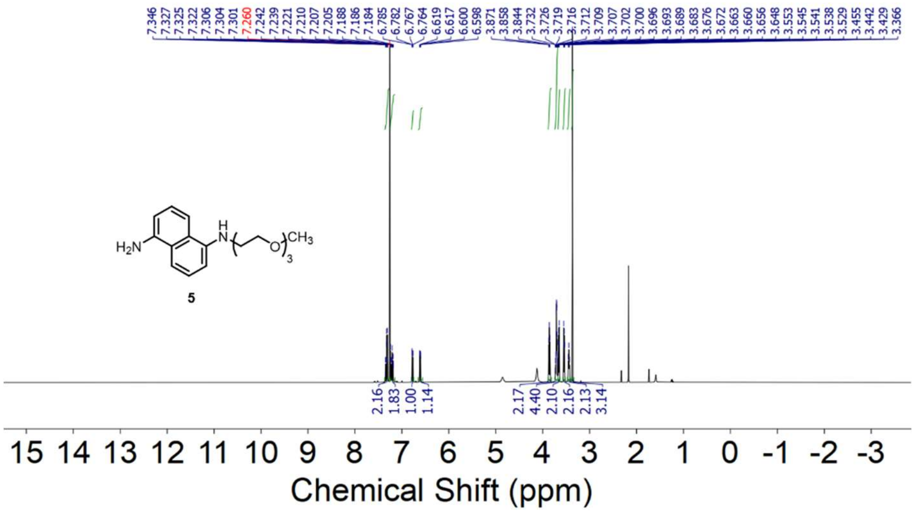

Figure S12. ^{1}H NMR spectrum (500 MHz, $CDCl_3$, 25 °C) of **5**.

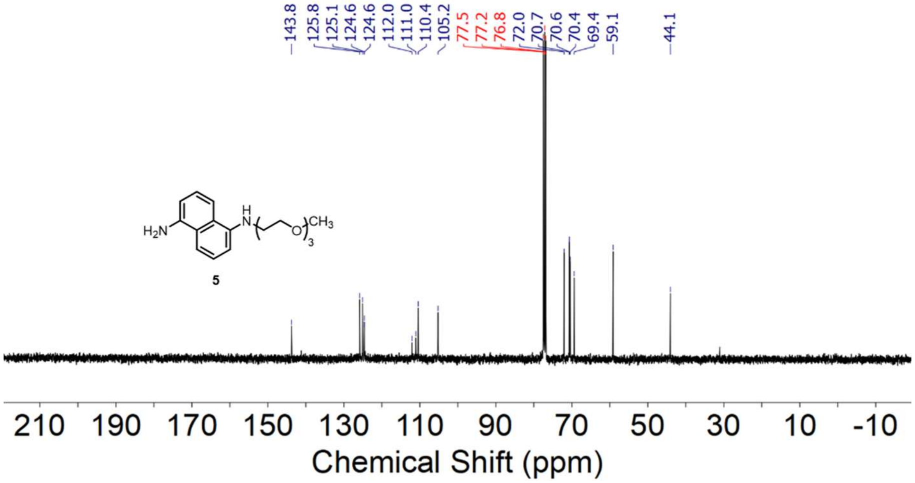

Figure S13. $^{13}C\{^{1}H\}$ NMR spectrum (100 MHz, $CDCl_3$, 25 °C) of **5**.

3. UV-vis Acid-Base Titration of Gc⊂CBPQT^{4+} Solutions

UV-vis spectra were measured during a titration of a 1:1 mixture of **CBPQT^{4+}** and guest **Gc** with an increasing number of equiv. of HCl and NaOH. This was done by preparing a 2 mL solution (3 mM) of **Gc⊂CBPQT^{4+}** in a mixture of ethanol/water (v/v = 3:1) in a quartz cuvette. 2 M HCl in H_2O was then added in from 0 up to 15 equiv., with UV-vis spectra measured after each addition. 2 M NaOH in H_2O was then added from 0 up to 15 equiv., with UV-vis spectra measured after each addition (Figure S14). For acid-base cycles under UV-vis measurement, each cycle was performed by adding 15 equiv. of HCl and 15 equiv. of NaOH, with UV-vis spectra measured after each addition.

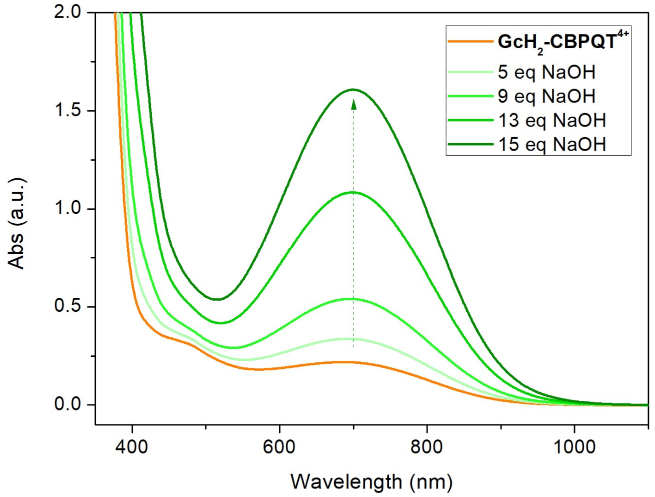

Figure S14. UV-vis titration of $\mathbf{GcH_2^{2+}{\subset}CBPQT^{4+}}$ in ethanol/water (v/v = 3:1) (3 mM). The addition of 2 M aqueous NaOH leads to deprotonation of the **DAN** unit, resulting in an increase in the intensity of the CT band at 700 nm.

The binding affinities of the **DAN** and **DOB** moieties were estimated by titration of 0.3 mM **Gc** with 50 mM $\mathbf{CBPQT^{4+}}$ solution and 3 mM $\mathbf{GcH_2^{2+}}$ (**Gc** with 30 equiv. HCl) solution with 100 mM $\mathbf{CBPQT^{4+}}$ solution, respectively; the titrations were monitored by UV-vis spectroscopy and were performed in triplicate. Figure S15 shows representative data. Absorbance at 700 nm wavelength in **Gc** titration and 474 nm wavelength in $\mathbf{GcH_2}$ titration was used to fit the isotherm of the corresponding complex. The titration reached completion at a 1:1 host-guest ratio. The resulting isotherms were fitted with a 1:1 global fitting model (Nelder-Mead method), using the BindFit platform.[1] Standard deviations were estimated from three replicates.

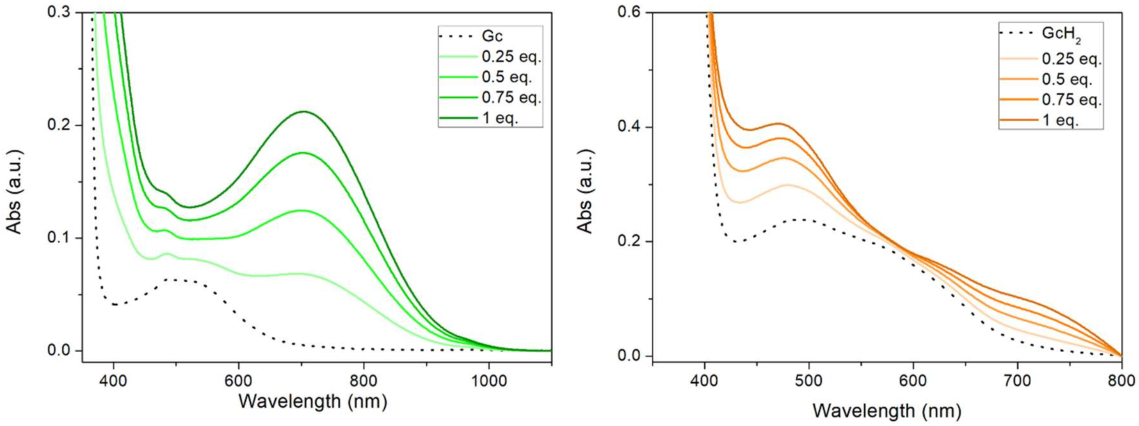

Figure S15. UV-vis spectra resulting from the titration of 0.3 mM **Gc** solution (left) and 3 mM $\mathbf{GcH_2}$ solution (right) with $\mathbf{CBPQT^{4+}}$ in ethanol/water (v/v = 3:1).

4. NOESY Spectrum of Gc⊂CBPQT^{4+} Solutions

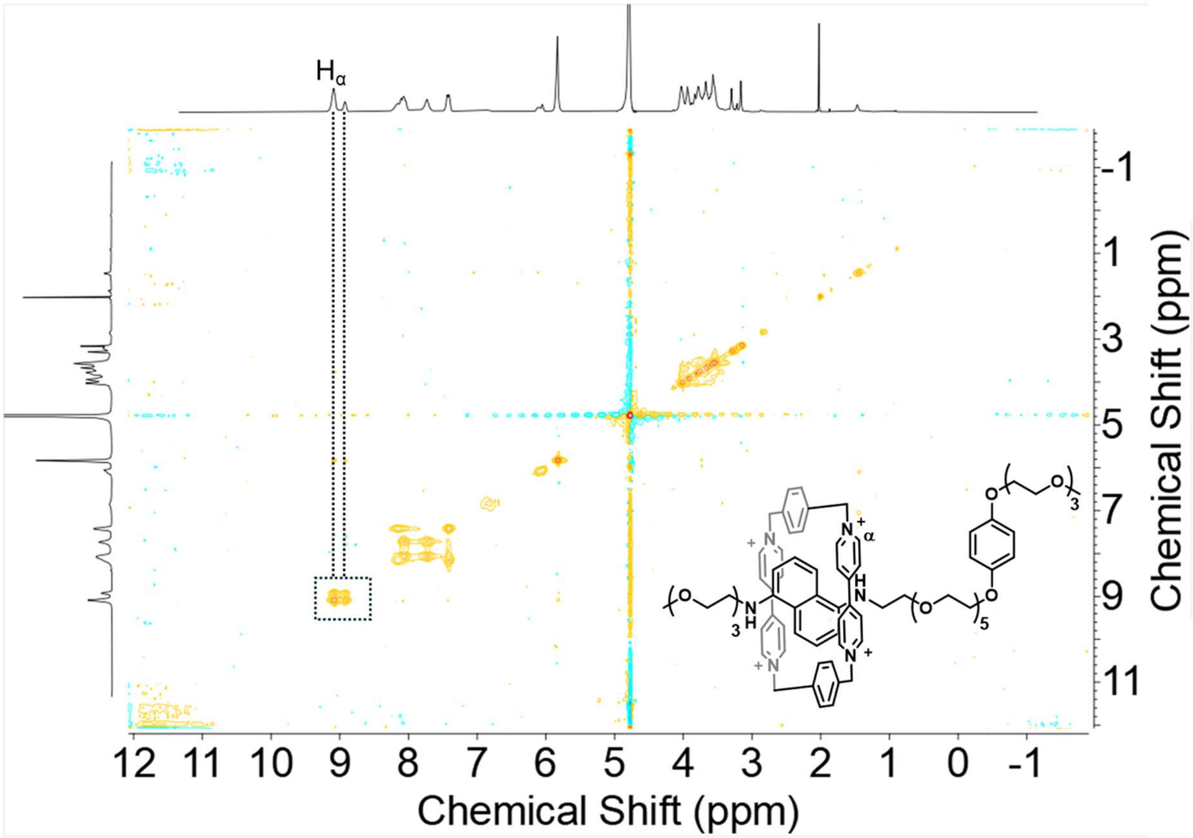

Figure S16. ^{1}H-^{1}H NOESY of 1:1 **Gc⊂CBPQT^{4+}** complex (600 MHz, D_2O, 25°C).

5. ^{1}H NMR Titrations of Guest⊂CBPQT^{4+} Solutions

^{1}H NMR titrations of **Guest⊂CBPQT^{4+}** complexes were recorded on **Gc** solutions in D_2O (500 μL, 3 mM). The guest started in either a neutral or protonated form (acidified with 30 equiv. of DCl to form **GcD$_2$**). Spectra were collected with increasing amounts of a [**CBPQT**]Cl_4 solution in D_2O (100 mM) added to the guest solution in the NMR tube. The number of equiv. of [**CBPQT**]Cl_4 used were as follows: 0, 0.25, 0.50, 0.75, 1.00, with the solution becoming increasingly green with each addition for the neutral complex, and increasingly pale orange for the acidified complex (Figure S17).

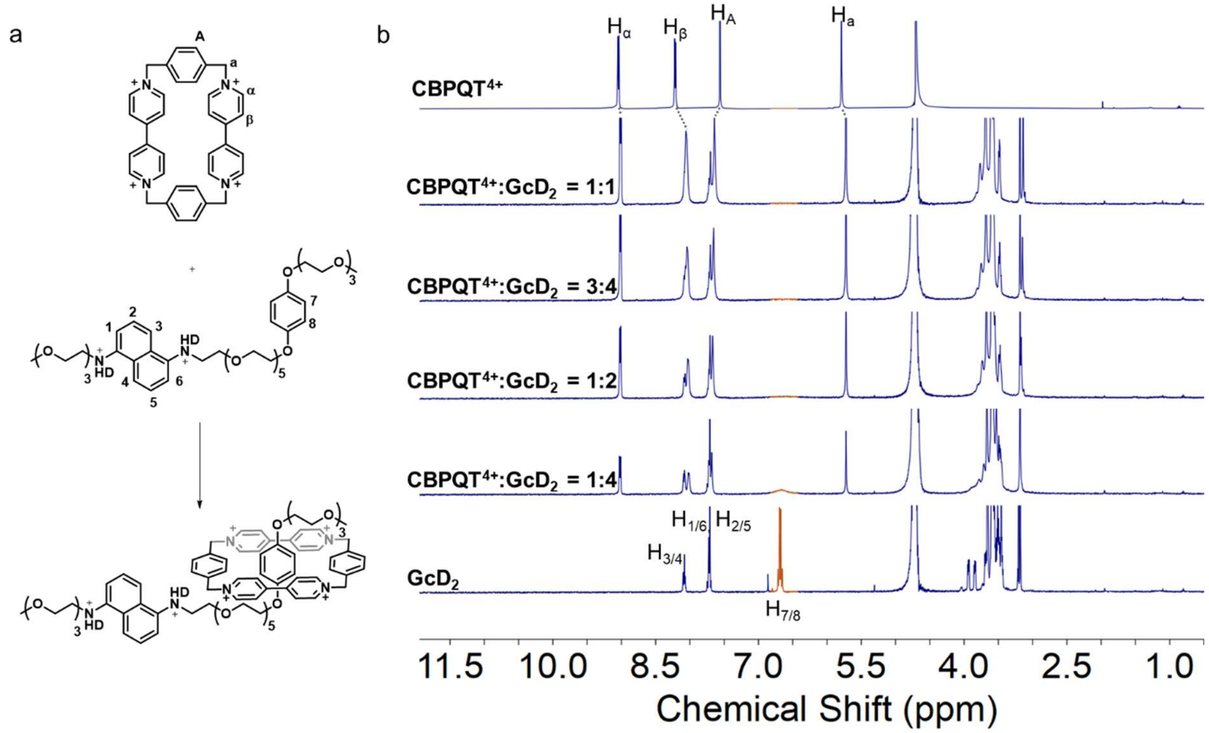

Figure S17. a) Illustration of the formation of $\mathbf{GcD_2}^{2+}\subset\mathbf{CBPQT}^{4+}$. b) ^{1}H NMR spectra (400 MHz, D_2O, 25°C) comparing $\mathbf{GcD_2}$, $\mathbf{GcD_2}^{2+}\subset\mathbf{CBPQT}^{4+}$ with different host-guest ratios, and $\mathbf{CBPQT}^{4+}$.

6. Acid-base switching of Gc⊂CBPQT^{4+} complex

Acid-base switching of $\mathbf{Gc}\subset\mathbf{CBPQT}^{4+}$ complex was performed by using DCl and DABCO as acid and base triggers. Increasing equiv. of DCl (2 M) in D_2O were added to the NMR tube used for the [**CBPQT**]Cl_4 titration from 0 to 15 equiv. The reverse process was studied by adding a solution of DABCO in D_2O (1 M) into a freshly prepared solution of $\mathbf{GcH_2}^{2+}\subset\mathbf{CBPQT}^{4+}$ in D_2O (500 μL, 3 mM, 30 equiv. DCl) from 0 to 30 equiv. The acid-base cycles were done by preparing a 400 μL, 3 mM $\mathbf{Gc}\subset\mathbf{CBPQT}^{4+}$ solution in D_2O, followed by adding 2 M DCl, measuring ^{1}H NMR spectra, adding 2 M DABCO and measuring ^{1}H NMR spectra again (Figure S18). The 1st cycle was performed by adding 15 equiv. of acid then 15 equiv. of base. Similarly, the 2nd cycle used 20 equiv. of each trigger, 3rd cycle used 25 equiv. of each trigger, 4th cycle used 30 equiv. of each trigger, and 5th cycle used 35 equiv. of each trigger.

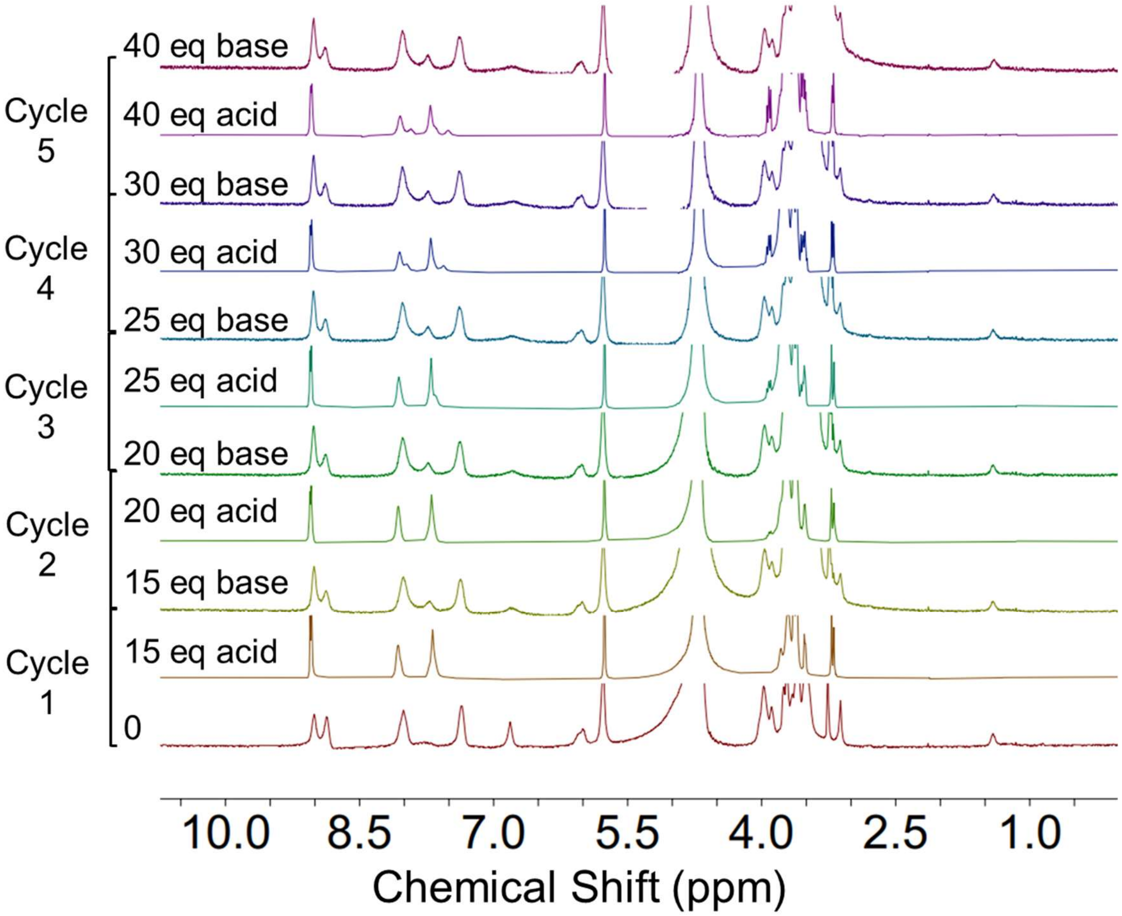

Figure S18. a) ^{1}H NMR spectra (400 MHz, D_2O, 25°C) of protonation and deprotonation of **Gc⊂CBPQT^{4+}** using aqueous DCl and DABCO solutions, respectively. Five cycles of switching between **Gc⊂CBPQT^{4+}** and **GcD$_2^{2+}$⊂CBPQT^{4+}** were monitored.

7. Preparation of CNC-CBPQT^{4+} Gels and Films

CNC-**CBPQT**$^{4+}$ gel: gels were assembled by mixing an aqueous CNC suspension (CNC-Na^+, 6.1 wt%, pH = 6.5) and a solution of [**CBPQT**]Cl_4 (100 mM in water). After shaking, ultrasonication (usually 5 mins) and standing overnight (18 h), the gel material was homogeneous and semi-transparent. The final concentration of **CBPQT**$^{4+}$ was set to 3.75 mM to achieve a 7 wt% CNC-**CBPQT**$^{4+}$ film as the final product.

CNC-**CBPQT**$^{4+}$ film: A colorless and transparent film was made by casting 1 mL 3.75 mM CNC-**CBPQT**$^{4+}$gel material on a polypropylene substrate (5 × 1 cm) and air-drying at room temperature overnight. This film contains 7 wt% host embedded and has a thickness of 86 μm.

8. Cycling of CNC-CBPQT^{4+} Films and UV-vis Analysis

The CNC-**CBPQT**$^{4+}$ films were infused with guest **Gc** by immersing the film in a solution of guest **Gc** in ethanol/water (v/v = 3:1) (20 mM, dark red) for 20 min. Afterward, the green film was removed from the solution and rinsed with ethanol/water (v/v = 3:1).

The CNC-**Gc**⊂**CBPQT**$^{4+}$ film was cycled by first immersing the film in a solution of HCl in ethanol/water (v/v = 3:1) (400 mM) for 2 min until the film was pale yellow. The film was then removed from solution and dried under air. Next, the film was immersed in a solution of ethanol/water (v/v = 3:1) for 15 min. The film was then removed from solution and dried under air. This cycling procedure was repeated for 5 cycles. UV-vis spectra of the film were collected at each step of the cycle.

9. Testing for the Presence of Leaked Guest Molecules

To determine whether the pseudorotaxane disassembles, the HCl and ethanol/water (v/v = 3:1) solutions that the CNC-**Gc**⊂**CBPQT**$^{4+}$ film was immersed in for each acid-solvent cycle were investigated by ^{1}H NMR spectroscopy. After removal of the film, the residual solutions were combined and evaporated. The residue was then dissolved in D_2O (500 μL), and the solution was transferred to an NMR tube, followed by ^{1}H NMR analysis (880 scans).

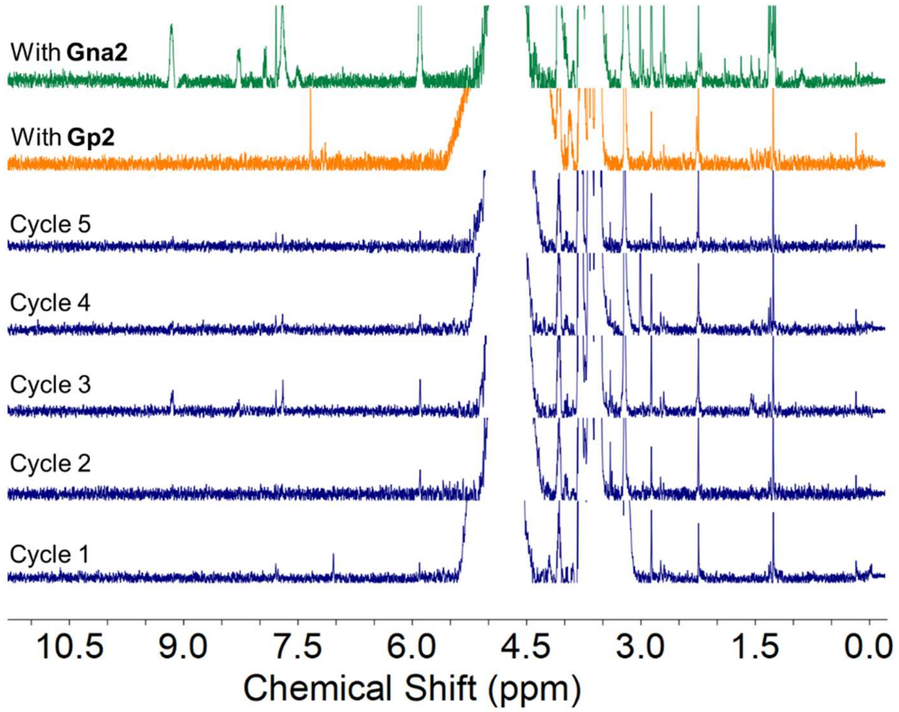

Figure S19. ^{1}H NMR spectra (300 MHz, 880 scans, D_2O, 25°C) of the leakage in acid-base treatment of CNC-**Gc⊂CBPQT^{4+}** film in each cycle and in control experiments with CNC-**Gp2⊂CBPQT^{4+}** film and CNC-**Gna2⊂CBPQT^{4+}** film.

10. Preparation and UV-vis Spectra of Pattern-Encryption Sample

Encryption of a selected area: 20 mM **Gc** solution (ethanol/water (v/v = 3:1)) was used as ink to selectively paint an area on a piece of 7 wt% CNC-**CBPQT^{4+}** film. Then the whole film was immersed in 400 mM HCl solution for 2 minutes. The colored area turned into pale orange.

Preparation of background area: 20 mM **Gp2** solution (ethanol/water (v/v = 3:1)) was used as ink to selectively paint the background area until both regions showed same color. During this process, it is necessary to try to prevent painting over encrypted area.

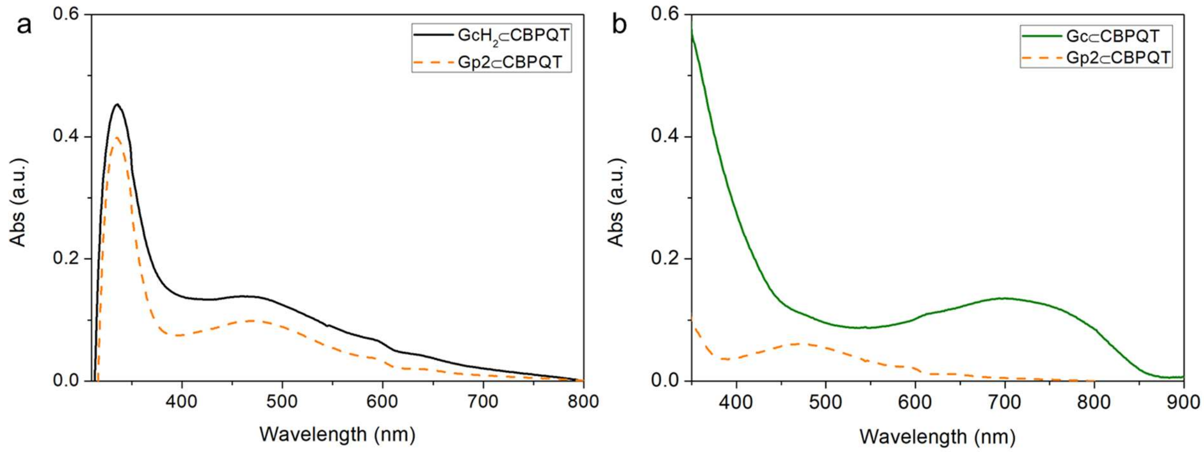

Figure S20. UV-vis spectra of a) encrypted area (**GcH_2^{2+}**) and background area (**Gp2**), and b) decrypted area (**Gc**) and background area (**Gp2**) in pattern-encryption sample.

Reference

[1] Supramolecular.org - Binding Constant Calculators. http://supramolecular.org (accessed 2024-10-04).
